# Association between COVID-19 and angiotensin-converting enzyme inhibitors with the spotlight on zinc: an opinion 

**DOI:** 10.1080/07853890.2021.1981545

**Published:** 2021-09-23

**Authors:** Bashar Khiatah, Amylee Amos, Deborah Carlson

**Affiliations:** a Department of Internal Medicine, Community Memorial Hospital, Ventura, CA, USA; b Amos Institute, Ventura, CA, USA; c Program Director, Internal Medicine Department, Community Memorial Hospital, Ventura, CA, USA

**Keywords:** COVID-19, SARS-CoV-2, Zinc, Critical Care, Nutrition, ACEI

## Abstract

In the setting of the raging COVID-19 pandemic, the search for innovative therapeutics is desperately sought after. As we learn more about the characteristics and metabolic health of patients and as our understanding of COVID-19 pathophysiology and treatment progresses, so is our understanding of medication effects that might increase disease severity. As of late, ACE inhibitors have been under investigation for a potential increase in illness severity due to ACE2 upregulation. Given our knowledge of other nutrient-pharmaceutical interactions, could the ACE inhibitor impact on COVID be due to something else? In this paper, we discuss the possibility that ACE inhibitors might be affecting COVID-19 patients by causing zinc insufficiency.

**KEY MESSAGES**
1. Zinc deficiency caused by chronic ACE inhibitor usage may exacerbate the pathogenicity of COVID-19 in susceptible patients.
2. A multi-center study is needed to assess the zinc levels of patients with COVID-19 who are taking ACE inhibitors and other medications that may result in low zinc levels.

## Introduction

SARS-CoV-2 (Severe acute respiratory syndrome coronavirus 2), the novel coronavirus that was first reported in Wuhan, China, in December 2019, has spread worldwide, causing coronavirus disease 2019 (COVID-19) pandemic. COVID-19 new cases and death numbers are on the rise globally with an estimated fatality rate of 0.5–1.0% [1–3]. Multiple areas where the COVID-19 pandemic hit hard have been suffering from critical shortages in supplies such as ventilators and intensive care unit (ICU) beds [4]. SARS-CoV-2 is the third zoonotic-human coronavirus, in which bats serve as the natural host, and with an undetermined intermediate reservoir [5]. This virus is positive-stranded RNA, enclosed by a protein-decorated lipid bilayer containing a single-stranded RNA genome and has an 82% homology with human SARS-CoV, which causes SARS (severe acute respiratory syndrome) [6].

Virus-bearing respiratory droplets, are the main transmission route of SARS-CoV-2 [7]. Patients usually develop symptoms within 5–6 days after exposure, with wide initial presentation variation from asymptomatic to severe illness, including acute respiratory distress syndrome (ARDS), multi-organ failure, and shock requiring ICU care [8]. Illness severity and death risk depend on the patient’s risk factors and underlying health issues, including male gender, advanced age, cardiovascular disease (CVD), types 1 and 2 diabetes mellitus, and obesity [9–10]. In all of these underlying health issues and others, nutrition status plays a significant role as a cause, treatment, and outcome determinant. This paper will focus on zinc in patients with SARS-CoV-2 who are on Angiotensin-Converting Enzyme inhibitor (ACEI).

## Discussion

### Zinc

Zinc is a trace element that plays an essential role in growth, reproductive health, neurobehavioral development, and immunity with potent immunoregulatory, antioxidant reactions, and antiviral properties [11]. Zinc also plays an essential role in the activity of many enzymes such as angiotensin-converting enzyme (ACE), carbonic anhydrases (CA), neutral endopeptidase (NEP) that regulates fluid homeostasis, acid-base balance, and vascular tone. The human body contains approximately 1.5–3.0 grams of zinc in a healthy individual [12]. Since zinc is essentially an intracellular ion, serum and plasma zinc levels do not necessarily reflect an accurate measure of the body’s zinc levels. Therefore, serum superoxide dismutase and erythrocyte alkaline phosphatase are utilised as an indirect marker of zinc status [13].

Food sources of zinc include animal foods such as oysters, liver, beef, and plant foods like legumes, nuts, and whole grains [12]. The absorption process occurs in the jejunum and to a lesser extent in the stomach and large intestine [14]. Absorption of zinc requires hydrolysis from amino acids before absorption. Zinc is absorbed through two mechanisms: carrier-mediated transport and diffusion. Assuming adequate daily zinc intake, about 7–9 mg of zinc is absorbed through the enterocyte per day, primarily through protein carrier Zrt- and Irt- like protein (ZIP)-4. Diffusion occurs paracellularly through the enterocyte when ZIP4 carrier absorption is at capacity, approximately 20 mg or more. High intakes of Zinc downregulate absorption and increase excretion, whereas suboptimal intakes increase ZIP4 synthesis [12].

In the zinc-adequate state, excretion happens mainly *via* the gastrointestinal tract and up to 10% through the urine. Other pathways for zinc excretion are not regulated, such as the growing out of hair, the desquamation of skin, and sweating [15]. From a nutritional standpoint, zinc absorption is enhanced by glutathione, organic acids including citric acid and picolinic acid, and in an acidic environment, and is decreased by phytic acid, oxalic acid, gallic acid, tannins, folate, iron, and calcium [12]. Pharmacologically, zinc levels are affected by the chronic use of drugs that affects either decreased absorption or increased secretion. Medications that decrease absorption are antacids, H2 blockers, and proton pump inhibitors (PPIs). Medications that increase zinc excretion are hydrochlorothiazide, furosemide, angiotensin receptor blockers (ARBs), and finally, with angiotensin-converting enzyme (ACE) inhibitors [13].

### ACEI

ACEI is responsible for blocking the conversion of multiple peptides in the Renin Angiotensin System (RAS) which primarily controls blood pressure and electrolyte balance and plays a role in cell differentiation, apoptosis, growth and to a certain extent inflammation [16]. Since the main entry receptor for SARS-CoV-2 is angiotensin-converting enzyme 2 (ACE2) [17], and ACE2 expression may be up-regulated by ACE inhibitors and ARBs [18], a huge scientific-pharmacological debate started about ACEI use in patients with infection or who are at high risk for severe SARS-CoV-2 infection [19,20]. These observations have led to speculation regarding the potentially harmful effects of ACE inhibitors and ARBs and a push for multiple clinical trials to assess the safety and address these concerns. None of these studies or articles have addressed that the ACEI effect on the SARS-CoV-2 severity might originate from their chronic use, causing subsequent underlying zinc deficiency. That being said, zinc antiviral activity and the effect of zinc deficiency have been well studied and reported in patients with SARS-CoV-2 infection [21]. The proposed antiviral properties of zinc include inhibition of RNA synthesis, topoisomerase, and viral replication [22]. Jothimani D.*et al.* reported in their small study a higher complication rate (70.4% vs 30.0%, *p* = .009) in zinc-deficient SARS-CoV-2 patients, significant trend towards the development of ARDS (18.5% vs 0%, *p* = .06), longer length of hospitalisation (mean 7.9 vs 5.7 days, *p* = .048), and increased mortality (5 (18.5%) vs 0 (0%), *p* = .06), all of which are indicative of a severe disease in these patients. This study reported that zinc-deficient patients would most likely have severe SARS-CoV-2 infection, more complications, and higher mortality, which was reiterated and reinforced by another study performed in Japan that resulted in recommending the administration of zinc salts for these patients [20]. It is worth mentioning that none of these studies have appropriately assessed the efficacy of administering oral zinc given the complex process of absorption required to absorb zinc, especially since many of these patients are already on medication or will require a medication that prohibits or decreases zinc absorption, with PPIs being one of the most commonly used.

## Conclusion

In summary, in the setting of the COVID-19 pandemic and the wide use of ACEI for multiple chronic diseases, it is clear that a multi-center study is needed to assess zinc levels in patients with SARS-CoV-2 infection who are on ACEI (and any other medicine that decreases zinc level). Additionally, we must increase the awareness of practicing physicians and dietitians of the importance of zinc levels and the possible need for temporary supplemental zinc support for patients with high risk for SARS-CoV-2 and or patients who are on ACEI.

## Author contributions

Bashar Khiatah M.D. is the primary author involved in the conception and design of this article, including drafting of the paper. Amylee Amos MS, RDN, IFMCP completed the description and analysis of zinc.

Deborah Carlson M.D. FACP revised this paper critically for intellectual content.

## Data Availability

Data sharing is not applicable to this article as no new data were created or analysed in this study.
